# Trigemino-Vagal Recalibration in Pediatric Anesthesia: A Prospective Cohort Study on the “60-Minute Autonomic Cliff,” “Trigger Mass,” and Recovery Dynamics in 1115 Dental Procedures

**DOI:** 10.3390/jcm15103606

**Published:** 2026-05-08

**Authors:** Gözde Nur Erkan

**Affiliations:** 1 Anesthesiology and Reanimation, Clinic of Anesthesiology, Department of Oral and Maxillofacial Surgery, Faculty of Dentistry, Kırıkkale University, 71450 Kırıkkale, Türkiye; gozdenurerkan@kku.edu.tr; 2 Department of Statistics, Faculty of Engineering and Natural Sciences, Kırıkkale University, 71450 Kırıkkale, Türkiye

**Keywords:** trigeminocardiac reflex, pediatric anesthesia, autonomic instability, dental rehabilitation, lingual manipulation, autonomic cliff

## Abstract

**Background**: The trigeminocardiac reflex (TCR) is a potent brainstem response often underrecognized during pediatric dental procedures. We aimed to quantify TCR dynamics, identifying procedural and temporal predictors of occurrence and resolution. **Methods**: We conducted a prospective observational study (NCT07240688) in pediatric patients undergoing dental procedures under standardized sevoflurane anesthesia. TCR was defined as a ≥10% (mild) or ≥20% (severe) abrupt decrease in heart rate (HR) and/or mean arterial blood pressure (MABP). Data were analyzed using Generalized Estimating Equations (GEE) and hierarchical logistic regression to identify procedural risk factors and recovery dynamics. **Results**: The study included 85 pediatric patients (aged 2–9 years) undergoing 1115 monitored dental procedures. The overall TCR incidence was 82.3% (*n* = 70). Operative duration was the strongest predictor of occurrence; each 1 min increase raised TCR odds by 6.7% (aOR: 1.067, *p* < 0.001). A landmark “60-min Autonomic Cliff” was identified: the probability of rapid spontaneous recovery dropped from 97.1% before 60 min to 0.0% thereafter (*p* < 0.001). Pulpal involvement was associated with a 3.37-fold increase in odds of severe TCR (Cramer’s V = 0.747). While lingual manipulation was strongly associated with rapid resolution (OR: 650.04), deep pulpal maneuvers led to a state of “Vagal Lock-in”—a sustained bradycardic response with reduced spontaneous recovery—effectively neutralized by atropine (97.0% success). **Conclusions**: Pediatric TCR is a time-dependent autonomic phenomenon in which operative duration influences reflex susceptibility and recovery dynamics, without affecting reflex severity. Beyond the “60-min Autonomic Cliff,” spontaneous recovery becomes unlikely, marking a transition to a refractory physiological state rather than an increase in reflex severity. This threshold provides a clinically actionable signal for anesthesiologists to intensify monitoring and consider early vagolytic intervention, supporting anticipatory, time-guided intraoperative management.

## 1. Introduction

The trigeminocardiac reflex (TCR) is a potent brainstem-mediated autonomic response characterized by abrupt bradycardia, asystole, and hypotension following stimulation of any branch of the trigeminal nerve. While extensively documented in neurosurgical and craniofacial interventions, TCR during routine dental procedures remains underrecognized, particularly in pediatric patients where autonomic instability is heightened under general anesthesia [1,2]. Importantly, TCR is primarily detected intraoperatively by anesthesiologists, highlighting the need for awareness of its incidence, recognition, and management in perioperative care [3]. From an anesthetic perspective, TCR constitutes a potentially life-threatening intraoperative event requiring prompt recognition and management. Despite its significance, recent bibliometric evidence reveals an “identification gap” in standard literature, where TCR events during routine dental treatments are frequently missed [4].

Several TCR subtypes—central, peripheral, and Gasserian ganglion-mediated—exhibit distinct hemodynamic profiles [5]. Traditionally, central TCR has been defined as a ≥20% decrease in heart rate (HR) and mean arterial blood pressure (MABP) in response to mechanical, electrical, or chemical stimulation of any trigeminal branch [1,6]. However, emerging evidence indicates that peripheral variants may produce subtler hemodynamic changes, sometimes below this threshold, highlighting the need for vigilance even during minor manipulations [2,5,7]. While central TCR reliably manifests with significant HR and MABP declines, peripheral subtypes often exhibit a broader spectrum of autonomic changes, occasionally without MABP decline, necessitating a modified threshold of ≥10% to capture clinically relevant events [5,8,9,10].

TCR can be triggered by major dental interventions such as implant surgery, orthognathic surgery or dental extractions, but may also occur in association with mechanical lingual manipulation, including tongue traction, elevation, and sublingual pressure as described in a previously published case report involving floor-of-mouth surgery [11,12,13,14,15]. This reflex is similarly well-characterized in neurosurgical and neurointerventional settings, where trigeminal stimulation during skull-base and cerebrovascular procedures frequently elicits comparable autonomic responses, underscoring its broader clinical relevance [16]. These stimuli can lead to sudden intraoperative instability even when baseline vital signs appear stable. Although triggered by dental manipulation, TCR is fundamentally a neuroanesthetic phenomenon. Increased awareness of these specific triggers is essential for timely intervention and enhanced patient safety [5,16].

TCR is typically reversible once the triggering stimulus is removed [5], yet the dynamics of reflex onset and recovery under general anesthesia—particularly in relation to dental procedure type, oral site, and lingual retraction—remain poorly characterized [13]. Pediatric patients are uniquely susceptible due to the heightened sensitivity of developing autonomic pathways and facilitatory effects of brainstem serotonergic circuits [17,18]. Consequently, the incidence of TCR during routine dental procedures may exceed estimates derived from adult populations, emphasizing the urgent need for prospective, systematic investigation [1,19].

This prospective study investigated 85 pediatric patients undergoing dental procedures under general anesthesia. We systematically monitored HR and MABP to identify predictors of TCR, classifying episodes as “mild” (≥10% decrease) or “severe” (≥20% decrease). By examining anesthesia duration, procedure type, location, and lingual manipulation, we aim to provide robust clinical data to inform anesthetic management and refine safety protocols for this underappreciated reflex.

## 2. Materials and Methods

### 2.1. Study Design and Ethical Oversight

This prospective observational study was conducted at Kırıkkale University, Faculty of Dentistry, Anesthesiology Clinic, between 13 October 2025, and 27 March 2026, following the Kırıkkale University Non-Interventional Research Ethics Committee approval (Decision No: 2025.09.04, Date: 24 September 2025). Written informed consent was obtained from the parents or legal guardians of all pediatric participants prior to inclusion in the study.

This study was conducted and reported in accordance with the STROBE (Strengthening the Reporting of Observational Studies in Epidemiology) guidelines, and a completed STROBE checklist is provided as Supplementary Materials. The study adhered to the Declaration of Helsinki. The study protocol was prospectively registered at ClinicalTrials.gov (NCT07240688).

The study was initially designed as a dual-cohort prospective observational study including both adult and pediatric patients (50 per group). Adult case accrual was limited due to institutional referral patterns and workflow organization for dental procedures under general anesthesia, resulting in a predominantly pediatric population during the study period. To ensure adequate statistical power and feasibility within the study period, the protocol was prospectively revised to include only pediatric patients.

This amendment was approved by the Institutional Ethics Committee (11 March 2026, Meeting No. 2026/04) and subsequently updated in the ClinicalTrials.gov registry (NCT07240688) to maintain methodological transparency prior to any outcome analysis. The decision was based solely on recruitment feasibility and was made independently of any interim data review; no study data were accessed or analyzed before protocol modification. No interim outcomes or distributional analyses were performed during recruitment. Following the amendment, adult participants were excluded from the final analysis, and pediatric recruitment continued prospectively until the predefined target sample size of 85 was reached. This sample size satisfied the more conservative of two a priori power calculations (N = 85 for multivariable regression vs. N = 68 for TCR incidence). Following the protocol amendment, sample size estimation was restricted to the pediatric-only cohort.

Sample size calculations were performed using two complementary approaches. First, for multivariable analysis of potential predictors—including age, sex, anesthesia-related factors, and dental procedure characteristics (procedure type and the presence or absence of lingual retraction or tongue-base pressure)—sample size was calculated using the ‘pwr’ package in R Statistical Software (version 4.5.2; R Foundation for Statistical Computing). Assuming a medium effect size (f^2^ = 0.15), *α* = 0.05, and power = 0.80, the minimum required sample size was estimated as N = 85.

Second, a proportion-based calculation was conducted to estimate the required pediatric sample size for detecting clinically relevant TCR-related hemodynamic responses (≥10% reduction in heart rate). While prior literature reports a TCR incidence of approximately 52% in adults [7], a higher incidence of 70% was projected for pediatric patients due to their increased clinical sensitivity. The corresponding Cohen’s h for this comparison was 0.34. This approach indicated that at least 68 participants would be required (*α* = 0.05, power = 0.80). The final sample size was therefore determined based on the larger of the two estimates to ensure adequate power for all planned analyses.

### 2.2. Participant Selection and Methodological Rigor

Consecutive pediatric patients (aged 2–18 years) with ASA physical status I–II and developmental percentiles (25th–75th) were eligible for inclusion. The study was designed to encompass the full pediatric age spectrum to ensure generalizability and avoid upper-age selection bias. No upper age restriction was applied during recruitment; however, no patients aged 10–18 years requiring general anesthesia for dental procedures were treated during the study period, reflecting the institutional referral pattern for this population.

To standardize the autonomic baseline as much as possible, inclusion was restricted to patients meeting institutional protocol criteria based on preoperative Modified Yale Preoperative Anxiety Scale (m-YPAS) assessment. The m-YPAS was used as a standardized institutional tool to ensure comparable preoperative anxiety levels across included patients. In our institution, preoperative anxiety was initially managed using standardized non-pharmacological behavioral techniques. Only patients who did not require pharmacological premedication according to institutional protocol and who were able to enter the operating room under behavioral management were included in the study cohort. General anesthesia indication was based on clinical treatment requirements and cooperation status rather than anxiety level alone.

- Exclusion Criteria: Cardiac arrhythmias, pacemakers, or medications affecting chronotropy, dromotropy, or inotropy; neurological disorders influencing the TCR arc; and any intraoperative requirement for local anesthetics (excluding standardized extraction protocols).
- Rigorous Control: Patients receiving opioids at any stage or local anesthetics for non-extraction procedures were strictly excluded to isolate “pure” trigeminal-induced autonomic responses. Only the first cases of the day were enrolled to minimize circadian and hydration-related autonomic variability.

**Figure 1:**
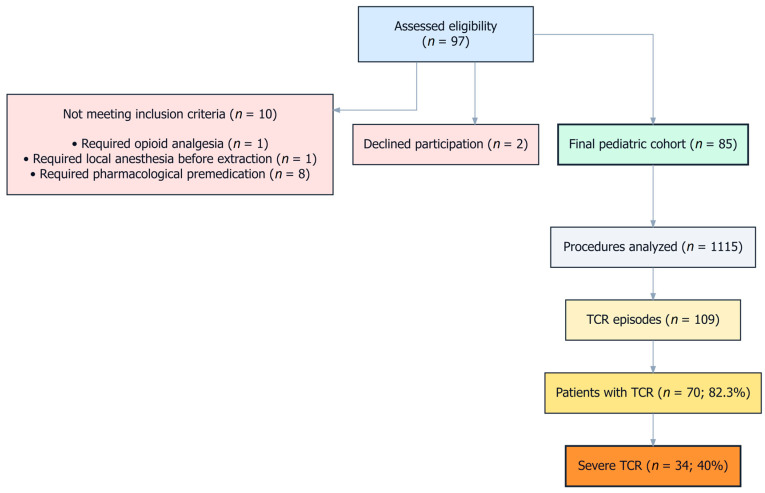
CONSORT-style flow diagram depicting patient screening, exclusion criteria, and final inclusion of pediatric patients in the study cohort. Abbreviations: GA, general anesthesia; TCR, trigeminocardiac reflex.

The study selection process, including exclusions and final cohort allocation, is presented in the CONSORT-style flow diagram (Figure 1).

### 2.3. Standardized Anesthesia and Respiratory Management

All patients were nasally intubated following a standardized protocol and received general anesthesia administered by a single experienced faculty anesthesiologist to ensure protocol consistency. Anesthesia was induced with propofol (3 mg/kg) (Propofol-PF 1%; Polifarma, Tekirdağ, Turkey) and rocuronium (0.6 mg/kg) (Muscoblock, 50 mg/5 mL; Polifarma, Tekirdağ, Turkey), and maintained with sevoflurane (Sevorane; AbbVie, North Chicago, IL, USA) at 1 MAC for all patients. Anesthesia was delivered using GE anesthesia machine (GE CoreStation 620, GE Healthcare, Chicago, IL, USA), with standardized monitoring in accordance with American Society of Anesthesiologists (ASA) standards and maintenance protocols [20]. Maintenance of anesthesia was performed using a low-flow technique (fresh gas flow: 1 L/min) with a medical air/oxygen mixture (FiO_2_: 0.60), ensuring stable inspiratory concentrations throughout the procedure.

- Ventilation Strategy: To eliminate confounding hypercapnic or hypoxic autonomic shifts, end-tidal carbon dioxide (EtCO_2_) was maintained between 26 and 30 mmHg to minimize hypercapnia-induced autonomic variability. Tidal volume was set at 6–8 mL/kg, with respiratory rates adjusted to maintain target EtCO_2_ within age-appropriate physiological limits.
- Hemodynamic Standardization: Hemodynamic monitoring for TCR included continuous electrocardiography (ECG) for heart rate (HR) and non-invasive blood pressure (NIBP) at 5 min intervals. During suspected TCR events, HR was recorded continuously and NIBP was triggered immediately to calculate mean arterial blood pressure (MABP).

Sevoflurane was reduced to 0.7–0.8 MAC 20 min prior to procedural end, with spontaneous respiration supported via SIMV-VC (synchronized intermittent mandatory ventilation, volume-controlled) mode. For postoperative analgesia, intravenous paracetamol (15 mg/kg) was administered approximately 20 min before the end of the procedure.

### 2.4. Dental Procedures

All dental procedures are routinely performed by a pediatric dentist specialist or a senior pediatric dentistry resident in the institution. Local anesthesia is routinely applied exclusively prior to tooth extractions so that ensure uniform analgesic management. Specifically, tooth extractions were performed under infiltration anesthesia using articaine hydrochloride and epinephrine (Ultraver DS; Haver Farma, Istanbul, Turkey) containing 80 mg articaine and 0.01819 mg epinephrine per 2 mL.

Dental procedures were categorized based on the anatomical site of intervention: anterior (including incisors and canines; FDI teeth #1–3 in each quadrant) and posterior (including molars; FDI teeth #4–6 in each quadrant), according to the Fédération Dentaire Internationale (FDI) World Dental Federation tooth numbering system. In addition, maxillary and mandibular distributions (corresponding to trigeminal V2 and V3 territories, respectively) were analyzed to evaluate the potential influence of trigeminal nerve branch anatomy.

### 2.5. TCR Definition and Clinical Phenotyping

The trigeminocardiac reflex (TCR) was defined as a sudden decrease in HR and/or MABP occurring within seconds, triggered by stimulation of the trigeminal nerve branches during dental procedures. Hemodynamic changes attributable to sympathetic responses from pain or recovery from neuromuscular blockade were not considered TCR, thereby preventing false-positive TCR detection based on baseline HR and blood pressure fluctuations.

- Mild TCR: 10–20% decrease in HR and/or MABP relative to baseline. If hemodynamic changes (HR and/or MABP) did not reach critical levels (≥20%), no pharmacological intervention was administered. The dental team was instructed to remove or adjust any lingual retraction or pressure on the tongue root.
- Severe TCR: ≥20% decrease in HR and/or MABP. During severe TCR episodes if lingual retraction or tongue root pressure present, the stimulus was removed or shifted to the tongue surface, and intravenous atropine (0.01 mg/kg) (Atropin Sülfat; Onfarma, Samsun, Turkey) was administered immediately. The administration of supplemental atropine or epinephrine following procedural cessation was pre-specified as a rescue intervention in cases of insufficient clinical response.

Recovery criterion and timing: Recovery time was recorded as the interval for HR to return to ≥90% of baseline. Dental procedures in this process continued without unnecessarily prolonging general anesthesia.

Episodes were categorized into three clinical phenotypes based on the presumed triggering mechanism and temporal response characteristics. This classification incorporates both etiological and severity-based dimensions. Given the inability to directly verify the exact micro-stimulus in all cases, this classification should be interpreted as an operational, inference-based framework rather than a definitive etiological categorization, intended to enhance clinical interpretability. This classification is intentionally non-orthogonal, with Types 1–2 reflecting presumed stimulus-related triggers and Type 3 representing a severity-based clinical endpoint.

- Type 1 (Lingual/Soft Tissue): Mild episodes presumed to be associated with lingual manipulation (tongue retraction or tongue-root pressure), characterized by resolution following cessation of the stimulus, in the absence of concurrent pulpal or direct dental intervention.
- Type 2 (Dental/Pulpal): Mild episodes occurring in the absence of lingual manipulation, or persisting despite discontinuation of lingual traction, suggesting a possible association with direct operative dental stimuli (e.g., pulpal irritation).
- Type 3 (Severe): Episodes characterized by profound hemodynamic instability (≥20% decrease from baseline heart rate) requiring immediate pharmacological intervention (atropine administration) and interruption of the dental procedure. Clinical stabilization was prioritized over real-time identification of the triggering stimulus. Although operative conditions, including potential lingual and dental stimuli, were reviewed after stabilization, a definitive etiological attribution could not be reliably established due to concurrent stimuli and the urgency of intervention. Therefore, this category reflects severity of autonomic response rather than exclusion of a specific etiological factor and clinically supersedes Types 1 and 2 in the presence of life-threatening hemodynamic instability.

This framework should be interpreted as an operational, non-orthogonal model for intraoperative decision-making rather than a strict mechanistic taxonomy.

### 2.6. Statistical Analysis

Data were analyzed using R software (version 4.5.3; R Foundation for Statistical Computing).

#### 2.6.1. Descriptive Statistics and Visualizations

The normality of continuous variables was assessed using the Shapiro–Wilk test and visual inspection of Q-Q plots. Descriptive statistics were presented as mean ± standard deviation (SD) for normally distributed data, and median [interquartile range, IQR] for non-normally distributed data. Categorical variables were expressed as frequencies and percentages (*n*, %). Inter-group comparisons for TCR (+) and TCR (−) cohorts were performed using the Independent Samples *t*-test, Mann–Whitney U test, or Fisher’s Exact test as appropriate. To evaluate the clinical magnitude of differences, Standardized Mean Differences (SMD) were calculated (Cohen’s d criteria: 0.2 small, 0.5 medium, 0.8 large).

#### 2.6.2. Regression and Generalized Modeling

Predictors of TCR occurrence were identified using a two-step hierarchical binary logistic regression (*n* = 85). To analyze the clinical dynamics within the TCR (+) cohort (*n* = 70), Generalized Estimating Equations (GEE) with an Exchangeable working correlation matrix were implemented. This approach accounted for the intra-subject dependency of multiple TCR episodes (N = 109 events; mean 1.55 episodes per patient), providing robust standard errors and unbiased Odds Ratios (OR) for resolution dynamics. The optimal model was selected based on the lowest Quasi-likelihood under the Independence Model Criterion (QIC). Interaction terms (Type × Timing) were included to evaluate the dynamic sensitization of the autonomic reflex.

#### 2.6.3. Ordinal and Severity Analysis

Ordinal logistic regression for TCR frequency (0, 1, and 2+ episodes) was conducted using the MASS package (polr function). Mild TCR episodes were further subdivided into two subgroups: isolated HR decrease and combined HR + MABP decrease, reflecting the presence or absence of associated blood pressure changes. Associations between procedural depth and TCR severity (Mild subgroups and Severe) were assessed using Cramer’s V to evaluate effect size, where values > 0.5 indicated a strong clinical correlation.

#### 2.6.4. Model Diagnostics and Effect Sizes

Multicollinearity was assessed using Generalized Variance Inflation Factors (GVIF) via the car package, with adjusted values (GVIF^1/(2xDf)^) < 2.5 considered acceptable. Odds Ratios were converted to standardized effect sizes (d_eq_) following the methodology described by Chen et al. (2010) [21]. Effect size magnitudes were interpreted according to Cohen’s criteria and the extended guidelines proposed by Sawilowsky (2009) [22], where 0.2 represents small, 0.5 medium, 0.8 large, 1.2 very large, and ≥2.0 huge effects.

Model fit and explanatory power were assessed using Nagelkerke Pseudo-R^2^, Akaike Information Criterion (AIC), and Area Under the Curve (AUC) analysis. To ensure model stability, variables with zero-cell frequency were excluded to prevent perfect separation bias. Statistical significance was set at a two-tailed *p* < 0.05.

High-resolution visualizations were generated at 300 DPI using the ggplot2 and patchwork packages to ensure publication-quality clarity.

No missing data were present in the final dataset for the variables included in the analyses.

## 3. Results

### 3.1. Baseline Characteristics and TCR Incidence

Patient demographics and TCR incidence are summarized in Table 1. A total of 85 pediatric patients (median age: 5 years (IQR: 4–6; range: 2–9)) underwent 1115 procedures (341 anterior, 774 posterior). The overall incidence of the TCR was 82.3% (*n* = 70), with a total of 109 discrete episodes recorded. Among these, severe TCR occurred in 34 patients, corresponding to 40% of the cohort. Patients experiencing TCR accounted for 84.3% (940/1115) of the total surgical workload.

Preliminary bivariate analysis indicated that while demographic factors such as age and gender were balanced between groups, total operative duration was significantly longer in the TCR (+) cohort (144.6 ± 32.7 min) compared to the TCR (−) group (106.0 ± 25.4 min; *p* < 0.001). This difference represented a large effect size (SMD = 1.320).

All severe TCR episodes were managed with atropine; no instances of atropine resistance or requirements for epinephrine were observed. Among 75 “Mild” TCR episodes, 41 (54.7%) occurred without concomitant MABP drops. In contrast, all but one severe TCR episode involved a significant decrease in MABP (≥20%).

A hierarchical logistic regression approach was subsequently employed to isolate independent procedural risk factors, allowing for a structured assessment of variables across different clinical dimensions.

### 3.2. Predictors of TCR Occurrence and Frequency

Prior to multivariable modeling, potential multicollinearity between independent variables—particularly operative duration and procedural counts—was assessed using Variance Inflation Factors (VIF). Across all models, VIF values remained below 2.7 (range: 1.08–2.67), confirming that each parameter contributed independently to the regression estimates.

Hierarchical logistic regression identified total operative duration as the strongest independent risk factor for TCR occurrence. In the final adjusted model, each one-min increase in surgical duration was associated with a 6.7% increase in the odds of TCR (aOR: 1.067, 95% CI: 1.031–1.114, *p* < 0.001). Clinically, this translates to a 91% increase in odds for every 10 min increment of operative time (1.067^10^ = 1.91) (Table 2).

The effect size of the predictive models was evaluated using Nagelkerke’s Pseudo-R^2^ to determine the variance explained by the clinical predictors (Table 2). The baseline model (Model 1: age, gender, duration) demonstrated a substantial effect size (Nagelkerke R^2^ = 0.351). Incorporating specific dental procedures (Model 2) increased the explanatory power to R^2^ = 0.402. Transitioning to Model 2 yielded an incremental explanatory gain of 5.1%. Although baseline factors—primarily operative duration—remained dominant, the final model accounted for 40.2% of the total variance in TCR occurrence. This represents a strong clinical effect size according to Cohen’s criteria. Notably, specific dental procedures provided no significant predictive power beyond operative duration, highlighting the cumulative impact of surgical stress (Table 2).

Ordinal logistic regression further confirmed that operative duration was the only significant predictor of increased TCR frequency (OR: 1.057, 95% CI: 1.035–1.082, *p* < 0.001), accounting for 46.6% of the variance (Nagelkerke R^2^ = 0.466, AIC = 165.70, df = 75). While the standardized effect size per min was numerically small (d_eq_ = 0.031), its cumulative impact was clinically substantial: for every 10 min increment in surgical time, the odds of experiencing a higher frequency of TCR episodes increased by 74% (1.057^10^~1.74) (Table 3A).

**Figure 2:**
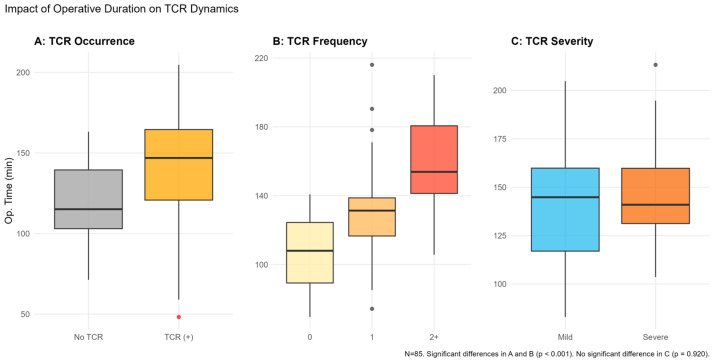
Boxplot analysis of operative duration across Trigemino-cardiac Reflex (TCR) clinical parameters. Distribution of total operation time (min) categorized by (**A**) TCR occurrence, (**B**) TCR frequency, and (**C**) TCR severity. (**A**) Occurrence: Operative duration was significantly longer in the TCR (+) group compared to the TCR (−) group (144.6 ± 32.7 min vs. 106.0 ± 25.4 min; *p* < 0.001). Multivariable logistic regression identified operative time as a robust independent predictor of TCR onset (aOR: 1.067, 95% CI: 1.03–1.11, *p* < 0.001). (**B**) Frequency: A dose–response-like relationship was observed between surgical duration and the number of TCR episodes (0, 1, and 2+). Ordinal logistic regression confirmed that each one-min increase in operative time significantly elevated the cumulative odds of experiencing higher TCR frequency (OR: 1.057, 95% CI: 1.035–1.082, *p* < 0.001), demonstrating a substantial effect size (Nagelkerke R^2^ = 0.466). (**C**) Severity: In contrast to occurrence and frequency, TCR severity (Mild vs. Severe) showed no statistically significant association with operative duration (*p* = 0.920). Clinical intensity appears to be independent of cumulative surgical stress. Horizontal lines within boxes represent the median; box limits indicate the interquartile range (IQR); whiskers represent the 1.5 × IQR. TCR: Trigemino-cardiac Reflex; aOR: Adjusted Odds Ratio; CI: Confidence Interval.

Operative duration was significantly longer in patients experiencing TCR compared to those without the reflex (Figure 2A). Furthermore, a clear dose–response relationship emerged between surgical time and TCR frequency, with longer procedures associated with an increased number of episodes (Figure 2B).

### 3.3. Analysis of TCR Severity

Subgroup analysis of TCR (+) patients (*n* = 70) revealed that neither age, gender, nor operative duration were independent predictor of severity (*p* > 0.05). However, female gender showed a borderline trend toward increased severity (OR = 2.63, 95% CI: 0.95–7.69, *p* = 0.068) with a medium effect size (d_eq_ = 0.53). The model showed robust fit and stability (AIC = 105.91, Residual Deviance = 89.91) (Table 3B). TCR severity did not appear to correlate with operative duration, suggesting that the intensity of the reflex is independent of cumulative surgical stress (Figure 2C).

A significant association was observed between dental procedure invasiveness and TCR severity (*p* = 0.028), with a Cramer’s V of 0.251 indicating a moderate effect size. Specifically, the incidence of ‘Severe’ TCR rose from 21.6% in superficial restorations to 48.5% during pulpal tissue manipulation. Risk analysis demonstrated that pulpal involvement was associated with a 3.37-fold increase in the odds of developing severe TCR compared to superficial treatments (95% CI: 1.19–9.94), representing a 237% increase in the odds (Table 4).

Regarding topographical distribution, the majority of severe TCR episodes (91.2%; 31/34) were localized in the posterior region (Table 3B). However, no significant difference in TCR incidence or severity was observed between maxillary and mandibular sites (*p* = 0.936, V = 0.035) (Table S1).

### 3.4. Predictors of TCR Resolution and Recovery Dynamics

**Figure 3:**
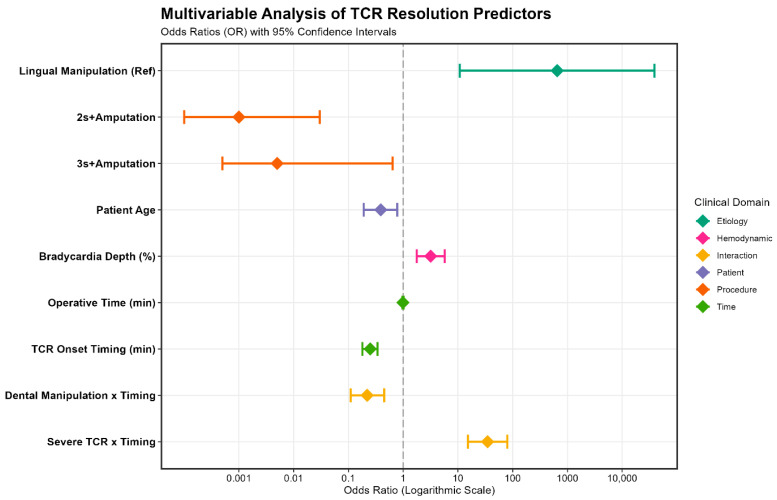
Forest Plot of Predictors for TCR Resolution Dynamics. Multivariable GEE (Generalized Estimating Equations) model visualizing the independent predictors of rapid versus delayed TCR resolution. Odds Ratios (OR) are presented on a logarithmic scale with 95% Confidence Intervals. Interpretation: OR values > 1 (right of the dashed vertical line) indicate factors that accelerate reflex recovery, whereas OR values < 1 (left) indicate factors associated with delayed resolution (prolonged recovery). Clinical Highlights: Lingual Manipulation showed the highest probability of rapid recovery (OR = 650.04). 2s/3s+Pulpotomy and Patient Age were significant predictors of delayed resolution (*p* < 0.05). The interaction terms (Dental Manipulation (Type 2) × Timing and Severe TCR (Type 3) × Timing) underscore the synergistic effect of procedural trauma and onset timing on the clinical course of the reflex. Note: Markers represent the point estimate (Diamond shape), and horizontal lines represent the 95% CI.

Multivariable GEE modeling identified complex posterior restorations with pulpal involvement—specifically two-surface and three-surface fillings combined with pulpotomy—as strong negative predictors of rapid recovery (OR: 0.001 and 0.005; d_eq_ > 2.9). Conversely, lingual manipulation predicted a powerful positive prognosis (d_eq_ = 3.57, *p* = 0.002) (Table 5A) (Figure 3).

**Table 5:** Multivariable GEE Models Evaluating Predictors of TCR Resolution: Impact of Interaction Effects, Anatomy, and Procedural Complexity (*n* = 109).

A. Multivariable GEE Model for Predictors of TCR Resolution with Interaction Analysis				
Predictor	Odds Ratio (95% CI)	*p*-Value	Effect Size (d_eq_)	Interpretation
Lingual Manip. (Type 1)	650.04 (10.8–38,942)	**0.002**	+3.57	Huge Positive
Type 2 × Timing	0.22 (0.11–0.45)	**<0.001**	−1.50	Sensitization
Type 3 × Timing	34.81 (15.2–79.8)	**<0.001**	+3.55	Synergistic
2s+Pulpotomy	0.001 (0.00–0.03)	**<0.001**	−3.85	Huge Negative
3s+Pulpotomy	0.005 (0.00–0.64)	**0.032**	−2.92	Huge Negative
Patient Age	0.39 (0.19–0.78)	**0.007**	−0.52	Medium-Large
Bradycardia Depth (%)	3.18 (1.77–5.72)	**<0.001**	+0.63	Medium-Large
Operative Time (min)	0.99 (0.97–1.02)	0.884	−0.001	Not Significant
TCR Onset Timing (min)	0.25 (0.18–0.34)	**<0.001**	−1.37	Significant
**B. Multivariable GEE Model for Anatomical and Procedural Predictors of TCR Resolution**				
**Predictor**	**Odds Ratio (95% CI)**	***p*** **-Value**	**Effect Size (d_eq_)**	**Clinical Magnitude**
Posterior vs. Anterior	8.29 (1.13–60.6)	**0.037** *	+1.17	Large
Mandible vs. Maxilla	1.69 (0.69–4.12)	0.248	+0.29	Small
2s+Pulpotomy	0.24 (0.04–1.57)	0.135	−0.80	Medium-Large
3s+Pulpotomy	0.35 (0.07–1.70)	0.193	−0.58	Medium

**Table 5A Notes:** Abbreviations: CI: Confidence Interval; OR: Odds Ratio; d_eq_: Equivalent Cohen’s d effect size; 2s+Pulpotomy: 2-surface restoration with pulpotomy; 3s+Pulpotomy: 3-surface restoration with pulpotomy. Clinical Definitions: Type 1: Lingual manipulation; Type 2: Dental procedure; Type 3: Severe TCR. Resolution: “Fast” is defined as ≤60 s. Statistical Interpretation: Odds Ratio (OR): Values > 1 indicate a higher probability of rapid resolution (positive prognosis), whereas OR < 1 indicates a higher risk of sustained TCR (negative prognosis). Significance: *p* < 0.05 was considered statistically significant. Bold *p*-values indicate statistical significance. All multivariate estimates were adjusted for subject-level clustering using Generalized Estimating Equations (GEE). **Table 5B Notes:** N = 109 episodes from 70 patients. Odds Ratios (OR) > 1 indicate a higher probability of rapid resolution (≤60 s). * The large effect size (d_eq_ = 1.17) and significant *p*-value for the posterior region confirm that anatomical localization is a primary independent predictor of autonomic recovery. Age was excluded from this model to prevent multicollinearity with surgical site variables. Bold *p*-values indicate statistical significance. Calculations: Odds Ratios are derived from exp (Estimate). Given the significance of the anatomical zone (*p* = 0.037), clinical significance was further assessed using Equivalent Cohen’s d (d_eq_) effect sizes. These were calculated via the Chinn (2000) [23] transformation formula: $d_{eq}=\frac{ln(OR)\cdot \sqrt{3}}{\pi }$.

### 3.5. Temporal Dynamics and Interaction Effects

A key finding was the significant interaction between TCR etiology and procedural timing (*p* < 0.001). In Type 2 (dental-induced) episodes, the probability of rapid resolution declined significantly as time from anesthesia induction elapsed (Interaction OR: 0.22). This temporal decay was absent in Type 1 (lingual-induced) cases, where recovery potential remained high regardless of timing (Table 5A).

In contrast, Type 3 (severe) episodes demonstrated a strong positive interaction with timing (interaction OR: 34.81). However, this finding should be interpreted with caution, as these episodes required immediate pharmacological intervention, thereby reflecting clinician-driven rescue effects rather than intrinsic recovery dynamics.

### 3.6. Topographical Influence on Recovery

Anatomical localization significantly influenced resolution speed (Table 5B). Posterior interventions showed a much higher probability of delayed resolution than anterior sites (*p* = 0.037, OR: 8.29, d_eq_ = 1.17), whereas jaw localization (maxilla vs. mandible) had no impact (*p* = 0.248). These results suggest the anterior–posterior axis, rather than the specific trigeminal branch (V2 vs. V3), is the primary determinant of reflex persistence.

### 3.7. The 60-Min Prognostic Threshold in Recovery Dynamics

A marked transition in TCR recovery was observed at the 60-min mark. During the first hour, the probability of Rapid Resolution remained remarkably high (100% for 0–30 min; 97.1% for 31–60 min). However, a prognostic collapse occurred immediately beyond this threshold; for all episodes occurring after 60 min, the rapid resolution rate dropped to 0.0% (*p* < 0.001), with 100% of cases exhibiting a Delayed Resolution profile. This shift identifies a definitive temporal boundary beyond which spontaneous autonomic recovery within the 60 s safety window becomes statistically improbable.

### 3.8. Efficacy of Pharmacological Intervention

Atropine (0.01 mg/kg) demonstrated exceptional efficacy in achieving definitive stabilization. In 97.0% (*n* = 32/33) of patients requiring intervention, a single dose achieved complete suppression of further TCR episodes. Only one patient required a second dose due to recurrence. This indicates that once muscarinic blockade is established, the trigemino-vagal arc is effectively neutralized for the remainder of the procedure.

## 4. Discussion

The present prospective study provides a robust, anesthesia-centered investigation of the TCR in pediatric patients. By quantifying perioperative hemodynamic responses during dental procedures, we demonstrate that TCR is not a rare, unpredictable event but a potentially life-threatening, time- and stimulus-dependent autonomic phenomenon. Operative duration, procedural invasiveness, and anatomical site emerge as critical predictors of risk, offering anesthesiologists a practical framework for anticipatory hemodynamic management under general anesthesia.

For clarity, we operationally define the three conceptual terms introduced in this study as follows: (1) “Trigger Mass” refers to the cumulative intensity of trigeminal stimulation required to elicit a clinically significant vagal response; (2) the “Autonomic Cliff” denotes a temporal threshold (approximately ≥60 min) beyond which spontaneous recovery of TCR episodes becomes unlikely, indicating a shift toward pharmacologically dependent resolution; and (3) “Vagal Lock-in” describes a sustained vagal-dominant state characterized by recurrent or persistent bradycardia requiring pharmacologic interruption, typically reversible with anticholinergic administration. These terms are proposed as study-specific operational concepts derived from the present dataset and are not established or universally accepted physiological definitions. Further studies are required to validate and generalize these constructs.

### 4.1. Beyond the “All-or-None” Paradigm: The “Trigger Mass” Effect

Our data challenge the conventional “all-or-none” perception of the reflex, revealing instead a graduated autonomic response precisely calibrated by surgical stimulus depth and what we term the “Trigger Mass.” We identified a distinct hemodynamic divergence: while superficial interventions rarely breached the mild threshold, pulpal involvement acted as a potent catalyst for severe, potentially life-threatening bradycardic surges (OR: 3.37).

This suggests a severity-dependent hemodynamic recruitment; while mild dental TCR may bypass the vasodepressor component—presenting as isolated heart rate deceleration (37.6% of events)—severe episodes almost universally involve systemic hypotension. For the clinician, this implies that the absence of early hypotension should not lead to complacency; a partial vagal response may serve as a silent precursor to profound instability.

Clinical Implications: Anesthesia providers should anticipate that the hemodynamic stability observed during superficial restorations does not guarantee safety during subsequent deep pulpal maneuvers. A “step-up” in monitoring vigilance is required the moment the pulp chamber is breached, regardless of prior stability.

### 4.2. The “Autonomic Cliff”: Temporal Sensitization and Kindling

A pivotal finding of our multivariable modeling is the profound impact of cumulative operative duration. Each one-min increase in surgery was associated with a 6.7% increase in TCR odds, translating to a 91% increase in odds for every 10 min increment. We hypothesize that this is driven by “neuro-vascular sensitization” and “kindling” mechanisms.

Kindling, a hallmark of neural plasticity, describes a process where repetitive sub-threshold stimuli eventually elicit a full-scale response [16,24]. This quantifiable escalation is consistent with “Temporal Summation,” suggesting the brainstem’s trigemino-vagal arc undergoes progressive functional instability [16,25,26]. Consequently, the surgical “timeline” is a dynamic risk factor; a maneuver tolerated in the 10th minute may precipitate a severe crisis in the 60th due to this underlying autonomic recalibration. This transition culminates in the “Autonomic Cliff”—a point beyond the 60-min mark where the probability of rapid spontaneous recovery drops to 0%.

Importantly, the ‘Autonomic Cliff’ should not be interpreted as an increase in TCR severity. Rather, it represents a transition to a refractory autonomic state characterized by loss of spontaneous recovery and increased dependence on pharmacological intervention. This distinction underscores that operative duration influences reflex susceptibility and recovery dynamics, whereas the magnitude of the hemodynamic response is primarily determined by stimulus characteristics.

Clinical Implications: The surgical “clock” should be viewed as a dynamic risk-stratification tool. As the procedure approaches the 60-min mark, the clinician should prepare for “refractory” bradycardic episodes that are unlikely to resolve with simple stimulus cessation, shifting the management strategy from passive observation to active pharmacological readiness.

### 4.3. Anatomical Topography: The Posterior High-Yield Zone

Our analysis provides a mechanistic resolution to previously “unclear” observations regarding anatomical triggers [7]. In our pediatric cohort, severe TCR risk was distinctly concentrated in the posterior (premolar/molar) region. This suggests that the density of afferent input from the molar region (Trigger Mass) is a more significant driver of the reflex than the anatomical pathway of the maxillary or mandibular nerves themselves. These findings suggest that the larger pulpal volume and complex, multi-canalled architecture of molars provide a superior substrate for both temporal and spatial summation within the trigeminal nuclei.

As illustrated in Figure 3, the interaction between procedural type and timing demonstrates a clear temporal sensitization pattern. Lingual manipulation (Type 1) maintains a rapid, stimulus-bound recovery regardless of timing, whereas pulpal stimuli (Type 2) exhibit progressive delay in resolution as operative time increases. This temporal sensitization highlights the cumulative autonomic burden of deeper dental procedures. Unlike the stimulus-bound, “on-off” binary response observed during lingual manipulation characterized by strong association, pulpal maneuvers exhibit a “non-binary” cumulative profile. While lingual triggers engage rapid-adapting Aβ fibers, pulpal stimuli likely engage slow-adapting C-fibers and inflammatory mediators, leading to sustained activation—a “wind-up” phenomenon that exhausts baroreceptor compensation [27,28,29].

The distinction between short- and long-latency components of the TCR provides a coherent neurophysiological framework for these observations. Short-latency responses are mediated by low-threshold, oligosynaptic pathways associated with non-nociceptive input, resulting in rapid onset and prompt recovery. In contrast, long-latency responses involve nociceptive, polysynaptic circuits, reflecting more complex brainstem processing and typically manifesting as more sustained and less reversible autonomic disturbances [30]. This framework may explain the divergent temporal and recovery characteristics observed between lingual manipulation–related and pulpal/dental-induced TCR episodes in our cohort.

In contrast, Type 3 (severe) episodes represent instances of profound autonomic instability that necessitate immediate pharmacological intervention. While their resolution occurs rapidly, this is largely driven by clinical rescue rather than intrinsic reflex dynamics. Consequently, the temporal sensitization patterns observed for Type 2 do not directly apply to Type 3, and their inclusion underscores the critical importance of early recognition and prompt management in the posterior high-yield zone. These multivariable GEE findings (Table 5A) empirically support the mechanistic distinctions outlined above, demonstrating that Type 1 (lingual) episodes resolve rapidly independent of timing, Type 2 (dental/pulpal) episodes exhibit temporal sensitization, and Type 3 (severe) episodes require immediate intervention, thereby validating the posterior high-yield zone as a critical locus of autonomic risk.

Clinical Implications: Clinically, this delineation informs intraoperative strategy: lingual-induced TCR may often be mitigated by simple cessation of traction, whereas pulpal-induced episodes, particularly in later stages of surgery, require vigilant hemodynamic monitoring and readiness for pharmacological intervention. This distinction is crucial for anesthesiologists in anticipating reflex resilience and optimizing patient safety. Procedural planning should explicitly account for this “posterior risk bias.” When the surgical team moves from anterior to posterior quadrants, the anesthesiologist should re-verify intravenous access and ensure that anticholinergic agents are immediately accessible, since the likelihood of a severe autonomic surge more than doubles in this region.

### 4.4. Neuroanatomical Pathways and Developmental Predisposition

The TCR is now better conceptualized within a distributed brainstem autonomic network rather than a linear reflex arc. Contemporary evidence highlights the nucleus tractus solitarius as a key integrative and modulatory hub within this system, contributing to the coordination of downstream vagal output via the nucleus ambiguus through complex medullary and supranuclear interactions [3,31,32].

While immaturity of the brainstem’s serotonergic systems renders children susceptible to exaggerated vagal surges [17,18], our model did not identify age as a significant predictor (*p* = 0.181). This suggests that under standardized 1.0 MAC sevoflurane anesthesia, the cumulative physiological stress—represented by operative duration—effectively overshadows developmental differences in autonomic maturation. Interestingly, the female gender showed a medium effect size for severity (d_eq_ = 0.53, OR = 2.63), aligning with evidence of higher baseline vagal reactivity in females [1,33]. This implies that while surgical factors dictate when a reflex occurs, the magnitude may be governed by intrinsic, gender-linked predispositions [16,34].

Clinical Implications: While pediatric patients are naturally predisposed to vagal surges, our findings suggest that procedural duration “levels the playing field.” Clinicians should treat every pediatric patient as “high-risk” for TCR once the 60-min threshold is crossed, irrespective of age or baseline maturity.

### 4.5. The “Extraction Paradox” and Pharmacological Neutralization

The relative absence of TCR during extractions—the “Extraction Paradox”—likely reflects the suppressive effect of late-stage sympathetic activation and the attenuation of the reflex arc by local anesthetic blocks [1,35,36]. Regarding management, our findings highlight a “Pharmacological Preconditioning” effect; patients receiving Atropine (0.01 mg/kg) early in the procedure exhibited zero TCR activity thereafter.

The observed 97% success rate of a single atropine dose in achieving total procedural silence is a remarkable finding. It suggests that muscarinic blockade does not merely manage the acute episode but effectively resets the sensitized trigemino-vagal arc, preventing the ‘kindling’ effect from precipitating recurrent crises despite ongoing surgical stimulation, consistent with a ‘Vagal Lock-in’–like state. Atropine acts as a potent “autonomic circuit-breaker.” While Pediatric Advanced Life Support (PALS) guidelines advocate for Epinephrine in refractory cases [35,37], our cohort’s effective management with atropine alone suggests that, in pediatric dental anesthesia, muscarinic blockade is often sufficient to override cumulative trigeminal sensitization.

Clinical Implications: The “Extraction Paradox” suggests that timing and local anesthesia may mask TCR. More importantly, the 97% success of atropine highlights that for pediatric dental TCR, a single dose often provides long-term “autonomic silence.” This justifies the early use of atropine in severe cases to prevent the repetitive cycle of “wind-up” and recurrent instability.

### 4.6. Pharmacological Modulation and Anesthetic Context in TCR

The relatively high incidence of TCR observed in this pediatric cohort (overall 82.3%, with 40% classified as severe) is consistent with the broader anesthesia and surgical literature when lower diagnostic thresholds and continuous intraoperative monitoring are applied, rather than representing an outlier specific to general anesthesia. In adult populations undergoing oral and maxillofacial surgery under local anesthesia, TCR-compatible bradycardia (≥10% heart rate reduction) has been reported in up to 70–80% of procedural phases, with clinically significant responses (>20%) occurring in approximately one-third of cases [38]. Similarly, in routine dental procedures under local anesthesia, TCR-related responses have been reported in approximately half of adult patients, although substantial variability exists across studies due to differences in diagnostic criteria, anesthetic techniques, and monitoring sensitivity [7]. In addition, transient bradycardia during intravenous sedation has been observed in nearly 18% of patients without hemodynamic instability [35], suggesting that subclinical vagal responses may be underrecognized in routine anesthetic practice.

The relatively high incidence of TCR in this cohort may reflect both enhanced detectability under standardized general anesthesia with continuous monitoring and increased autonomic susceptibility in pediatric patients due to immature regulatory control [1,2]. From a monitoring perspective, TCR appears to represent a continuum of trigeminally mediated autonomic responses, with observed incidence strongly influenced by detection sensitivity and diagnostic thresholds rather than anesthetic modality alone.

Across anesthetic conditions, including general and regional anesthesia, increasing anesthetic depth modulates the hemodynamic expression of TCR without preventing reflex initiation [39,40,41,42]. Pharmacologic vagolysis with atropine consistently attenuates bradycardic responses, underscoring the central role of vagal efferent pathways in TCR expression [43]. Within this framework, the present findings suggest that the observed incidence under sevoflurane-based anesthesia is more likely related to enhanced detection conditions rather than increased intrinsic susceptibility. Sevoflurane may contribute to autonomic modulation but does not abolish trigeminovagal reflex pathways [44].

Although opioid-free anesthesia might be expected to reduce trigeminovagal reflex expression due to known modulatory effects of opioids [41], the persistently high incidence observed in this cohort suggests that pharmacologic factors alone do not fully explain the reflex burden. Overall, these findings support the interpretation that TCR expression is primarily stimulus-driven, while anesthetic agents and perioperative monitoring conditions act as modulatory factors influencing its clinical detectability rather than its intrinsic incidence.

Importantly, this study provides one of the few prospective pediatric characterizations of TCR that extends beyond incidence reporting by integrating stimulus type, procedural timing, and reflex recovery dynamics under standardized sevoflurane-based general anesthesia. Unlike prior studies focusing on isolated surgical contexts or single time-point assessments, the present analysis captures the dynamic temporal behavior of TCR within a continuous monitoring framework.

To our knowledge, this is among the first prospective pediatric anesthesia studies to integrate TCR initiation, temporal evolution, and recovery kinetics within a single standardized cohort, together with pharmacologic response profiling. These findings support a clinically relevant interpretation in which TCR expression is dynamically influenced by procedural phase and stimulus intensity, while anesthetic depth and agent selection act as secondary modulators. From a clinical management perspective, the data support anticipatory vigilance during high-risk procedural phases and reinforce the effectiveness of early anticholinergic administration in maintaining cardiovascular stability during trigeminal stimulation.

Clinical Implications: From a clinical safety perspective, differences between general and local anesthesia should be interpreted in terms of detection conditions rather than intrinsic risk. Continuous monitoring under general anesthesia increases the visibility of transient autonomic fluctuations, whereas such responses may be less consistently detected under local anesthesia due to patient-related and procedural variability. Accordingly, TCR may be considered a trigeminal reflex with variable detectability across anesthetic techniques rather than a modality-specific risk phenomenon. Additionally, despite the absence of intraoperative opioid administration, the observed high incidence of TCR suggests that pharmacologic modulation alone is insufficient to explain reflex expression, while sevoflurane-based anesthesia may have contributed to autonomic modulation and increased detectability. However, more robust conclusions regarding these associations require further large-scale prospective studies.

### 4.7. Strengths and Limitations

The primary strength of this investigation lies in its rigorous prospective design and the application of Generalized Estimating Equations (GEE) to quantify longitudinal dynamics. By introducing the concepts of the “Autonomic Cliff” and “Trigger Mass,” we provide a novel prognostic framework. However, the lack of objective depth-of-anesthesia monitoring (e.g., BIS) and the single-center nature of the study are acknowledged. Future multicenter trials incorporating real-time EEG-based monitoring are warranted to validate the “Autonomic Cliff” across broader populations.

## 5. Conclusions

This study establishes a detailed neuro-anatomical framework to redefine the TCR as a quantifiable and clinically relevant hemodynamic phenomenon. We identify a fundamental divergence between a stimulus-bound, rapidly reversible response during lingual maneuvers and a time-dependent, progressively refractory state associated with invasive pulpal procedures.

Importantly, the 60-min “Autonomic Cliff” does not reflect an increase in TCR severity, but rather marks a transition toward reduced spontaneous recovery and increased dependence on pharmacological intervention. This finding indicates that operative duration governs reflex susceptibility and recovery dynamics, whereas the magnitude of the hemodynamic response is primarily determined by stimulus characteristics.

At this juncture, clinicians should transition from watchful waiting to proactive intervention. The administration of Atropine (0.01 mg/kg) serves as an effective “autonomic circuit-breaker,” ensuring procedural continuity.

By bridging brainstem neurophysiology with real-time outcomes, our proposed time-contingent protocol provides a critical guide to enhance intraoperative safety during complex pediatric dental interventions. Given the heightened autonomic responses observed in pediatric patients, our study reinforces the need for interdisciplinary collaboration between pediatric dentists and anesthesiologists to ensure patient safety during procedures with potential trigeminal stimulation.

## Figures and Tables

**Table 1:** Demographic and Clinical Characteristics of the Study Population and Distribution of TCR Events.

A. Demographic and Clinical Characteristics of the Study Population (N = 85)					
Variable	Total (N = 85) [Total Count]	TCR (−) (*n* = 15) [Median/Count]	TCR (+) (*n* = 70) [Median/Count]	*p*-Value	*SMD*
**Demographics**					
Age (years), med [IQR]	5.0 [4.0, 6.0]	5.0 [4.0, 6.0]	5.0 [4.0, 6.0]	0.367 ^1^	0.240
Gender (Female), *n* (%)	47 (55.3%)	8 (53.3%)	39 (55.7%)	1.000 ^2^	0.048
**Operative Data**					
Op. Time (min), mean ± SD	137.8 ± 34.9	106.0 ± 25.4	144.6 ± 32.7	**<0.001 ^3^**	1.320
**Anterior Region**	(*n* = 341) *				
- Filling, med [IQR]	3.0 [1.0, 5.0] (255)	2.0 [0.0, 4.0] (32)	3.0 [1.0, 5.0] (223)	0.093 ^1^	0.426
- Pulpotomy, med [IQR]	0.0 [0.0, 0.0] (11)	0.0 [0.0, 0.0] (1)	0.0 [0.0, 0.0] (10)	0.574 ^1^	0.216
- Extraction, med [IQR]	0.0 [0.0, 1.0] (75)	0.0 [0.0, 0.5] (7)	0.0 [0.0, 2.0] (68)	0.307 ^1^	0.409
**Posterior Region**	(*n* = 774) *				
- Filling, med [IQR]	6.0 [5.0, 7.0] (514)	7.0 [5.0, 7.0] (90)	6.0 [5.0, 7.0] (424)	0.991 ^1^	0.031
- Pulpotomy, med [IQR]	1.0 [0.0, 2.0] (105)	0.0 [0.0, 1.0] (10)	1.0 [0.0, 2.0] (95)	0.060 ^1^	0.595
- Extraction, med [IQR]	1.0 [1.0, 3.0] (155)	1.0 [1.0, 2.5] (29)	1.0 [1.0, 3.0] (126)	0.620 ^1^	0.100
**B. Clinical and Demographic Distribution of TCR Events (*n* = 109)**					
**Parameter**			***n* (%)** **or Mean ± SD [Range]**		
**Anatomical Distribution**					
Anterior			14 (12.8%)		
Posterior			95 (87.2%)		
Maxilla			55 (50.5%)		
Mandible			54 (49.5%)		
**TCR Severity Classification**					
Mild (Isolated HR drop)			41 (37.6%)		
Mild (Combined HR + MABP drop)			34 (31.2%)		
Severe (HR drop ≥ 20%)			34 (31.2%)		
**Recovery Characteristics**					
Recovery Time (seconds)			102.43 ± 100.73		

Notes: Continuous variables are presented as mean ± SD or median [IQR] based on the Shapiro–Wilk normality test. Categorical variables are presented as *n* (%). Total procedural counts are provided in parentheses (*n*). Statistical significance (*p* < 0.05) was determined using the: ^1^ Mann–Whitney U test (non-parametric continuous data); ^2^ Fisher’s Exact test (categorical data); ^3^ Independent samples *t*-test (parametric continuous data). Bold *p*-values indicate statistical significance. SMD (Standardized Mean Difference): Calculated to evaluate the magnitude of differences between the TCR (−) and TCR (+) groups regardless of sample size. *SMD* values were interpreted based on Cohen’s criteria: 0.2 (small), 0.5 (medium), and 0.8 (large) effect sizes. An SMD > 0.1 was considered to indicate a meaningful clinical imbalance between the groups, highlighting operative duration and premolar pulpotomy as key differentiating factors. * Indicates the cumulative number of procedures in the respective anatomical region (Total procedures = 1115).

**Table 2:** Hierarchical Logistic Regression Models for Predicting TCR Occurrence (N = 85).

Predictor Variables	Model 1: Baseline Factors aOR (95% CI)	*p*-Value	Model 2: Full Clinical Model aOR (95% CI)	*p*-Value
Demographics				
Age (years)	0.93 (0.60–1.43)	0.732	0.80 (0.48–1.30)	0.365
Gender (Female vs. Male)	1.67 (0.43–6.77)	0.456	1.99 (0.48–9.01)	0.347
**Operative Factor**				
Operation Time (min)	1.05 (1.03–1.09)	**<0.001**	1.067 (1.03–1.11)	**<0.001**
**Dental Procedures**				
Anterior Filling	-	-	0.71 (0.46–1.06)	0.094
Anterior Pulpotomy	-	-	2.08 (0.29–51.6)	0.552
Posterior Filling	-	-	1.03 (0.69–1.57)	0.892
Posterior Pulpotomy	-	-	1.15 (0.58–2.63)	0.707
**Model Diagnostics**				
AIC	66.87		71.47	
Residual Deviance	58.87		55.47	
Likelihood Ratio Test	-	-	*χ*^2^ = 3.40	*p* = 0.493

Abbreviations: aOR: Adjusted Odds Ratio; CI: Confidence Interval; AIC: Akaike Information Criterion; AUC: Area Under the Curve; TCR: Trigemino-Cardiac Reflex. Statistical Methodology: A two-step hierarchical (block-entry) binary logistic regression was performed to identify independent predictors of TCR. Model 1 (Baseline): Included non-modifiable patient factors (age, gender) and the primary surrogate for cumulative surgical stress (total operative duration). Model 2 (Full Clinical): Evaluated the incremental predictive power of specific dental procedures after adjusting for Model 1 variables. Bold *p*-values indicate statistical significance.

**Table 3:** Predictors of TCR Frequency and Severity: Ordinal and Multivariate Logistic Regression Analysis.

	A. Ordinal Logistic Regression Analysis for TCR Frequency (0, 1, and 2+ Episodes)					B. Multivariate Logistic Regression for TCR Severity (Mild vs. Severe)				
Predictor	Odds Ratio (OR)	95% CI	*p*-Value	Effect Size (d_eq_)	Magnitude	Odds Ratio (OR)	95% CI	*p*-Value	Effect Size (d_eq_)	Magnitude
Age (years)	0.92	(0.66–1.28)	0.627	0.04	Negligible	1.33	(0.92–1.98)	0.144	0.16	Small
Gender (Female)	0.95	(0.38–2.37)	0.920	0.03	Negligible	2.63	(0.95–7.69)	0.068	0.53	Medium
Operation Time (min)	1.057	(1.035–1.082)	**<0.001**	0.031	Negligible	1.001	(0.98–1.03)	0.920	0.00	Negligible
Anterior Filling	0.83	(0.63–1.09)	0.167	−0.11	Negligible	0.95	(0.67–1.35)	0.789	0.03	Negligible
Anterior Pulpotomy	1.76	(0.54–6.27)	0.355	0.31	Small	2.01	(0.56–8.48)	0.293	0.39	Small
Posterior Filling	0.90	(0.69–1.17)	0.441	−0.06	Negligible	0.99	(0.72–1.36)	0.933	0.01	Negligible
Posterior Pulpotomy	1.04	(0.71–1.51)	0.841	0.02	Negligible	0.93	(0.62–1.38)	0.722	0.04	Negligible

**Table 3A Notes:** Results are based on an Ordinal Logistic Regression (Proportional Odds Model) (N = 85). The dependent variable represents TCR frequency categories: 0, 1, and 2+ episodes (cases with 3 episodes were pooled into the 2+ category due to low frequency, *n* = 2). OR: Odds Ratio; CI: Confidence Interval; d_eq_: Standardized effect size. Model Fit and Explanatory Power: The ordinal logistic regression model demonstrated strong explanatory power with a Nagelkerke R^2^ of 0.466, indicating that 46.6% of the total variance in TCR frequency is accounted for by the included predictors. (AIC = 165.7; Residual Deviance = 145.7; df = 75). Multicollinearity: Variance Inflation Factors (VIF) were assessed to ensure the independence of predictors. All VIF values were below 2.6 (range: 1.09 to 2.54), indicating no significant inter-correlation between operative duration and procedural complexity. Bold *p*-values indicate statistical significance. **Table 3B Notes:** Results are based on a Multivariable Binary Logistic Regression conducted specifically within the cohort of patients who experienced at least one TCR event (*n* = 70). The dependent variable is TCR Severity (Mild: episode/s, defined as ≥10% drop from baseline heart rate with or without ≥10% drop baseline blood pressure; Severe: At least one Severe episode, defined as ≥20% drop from baseline heart rate). OR: Odds Ratio; CI: Confidence Interval; d_eq_: Standardized effect size. Model Fit and Stability: The model demonstrated stable parameters with a Residual Deviance of 89.91 and AIC of 105.91 (df = 62). Methodological Refinement: Multicollinearity: Potential collinearity within the subgroup (*n* = 70) was evaluated via Variance Inflation Factors (VIF). All values remained below 2.7 (range: 1.08 to 2.67), confirming the independence of predictors in the severity model. Bold *p*-values indicate statistical significance.

**Table 4:** Severity Grading and Risk Analysis of TCR Based on Procedural Depth (*n* = 109).

Procedure Group	Mild TCR (*n*, %) *	Severe TCR (*n*, %) **	Odds Ratio (95% CI)	*p*-Value	Effect Size (Cramer’s V)
1 or 2-S Fillings	40 (78.4%)	11 (21.6%)	1.00 (Ref)	**0.028**	0.251 (Moderate)
3-S Filling/SSC	18 (72.0%)	7 (28.0%)	1.41 (0.47–4.21)		
Pulpotomy	17 (51.5%)	16 (48.5%)	3.37 (1.19–9.94)		
Total	75 (68.8%)	34 (31.2%)			

* Mild TCR: Heart rate (HR) drop of 10–20% from baseline. ** Severe TCR: Heart rate (HR) drop of ≥20% from baseline. Abbreviations: SSC: Stainless Steel Crown; OR: Odds Ratio; CI: Confidence Interval; 1-S Filling: 1 surface restoration; 2-S Filling: 2 surface restoration; 3-S Filling: 3 surface restoration. Bold *p*-values indicate statistical significance.

## Data Availability

The data sets used and analyzed during the current study are available from the corresponding author on reasonable request.

## Supplementary Materials

The following supporting information can be downloaded at: https://www.mdpi.com/article/10.3390/jcm15103606/s1, Table S1: Statistical analysis of TCR severity across anatomical sites (*n* = 109); STROBE Statement Checklist: The study-specific completed checklist based on the STROBE guidelines is provided.

## Abbreviations

The following abbreviations are used in this manuscript:

BIS	Bispectral Index
BP	Blood Pressure
EtCO_2_	End-Tidal Carbon Dioxide
FDI	Fédération Dentaire Internationale
FiO_2_	Fraction of Inspired Oxygen
GEE	Generalized Estimating Equations
HR	Heart Rate
MAC	Minimum Alveolar Concentration
MABP	Mean Arterial Blood Pressure
NA	Nucleus Ambiguus
OR	Odds Ratio
PALS	Pediatric Advanced Life Support
TCR	Trigeminocardiac Reflex
