# Supplementary material for: Trigemino-Vagal Recalibration in Pediatric Anesthesia: A Prospective Cohort Study on the “60-Minute Autonomic Cliff,” “Trigger Mass,” and Recovery Dynamics in 1115 Dental Procedures
Source: J Clin Med. 2026 May 8;15(10):3606. doi: 10.3390/jcm15103606 (PMC13207552; doi:10.3390/jcm15103606)
Supplement: Supplementary file 1 [file jcm-15-03606-s001.zip › Supplementary_Material_STROBE-checklist-TCR.pdf]

STROBE Statement—checklist of items that should be included in reports of observational studies

	Item No.	Recommendation	Page No.	Relevant text from manuscript
**Title and abstract**	1	(*a*) Indicate the study's design with a commonly used term in the title or the abstract	Title/Abstract	**Trigemino-Vagal Recalibration in Pediatric Anesthesia: A Pro-spective Cohort Study on the "60-Minute Autonomic Cliff," "Trigger Mass," and Recovery Dynamics in 1,115 Dental Pro-cedures** We conducted a prospective observational study (NCT07240688) in pediatric patients undergoing dental procedures under standardized sevoflurane anesthesia.
		(*b*) Provide in the abstract an informative and balanced summary of what was done and what was found	Abstract	The study included 85 pediatric patients (aged 2–9 years) undergoing 1,115 monitored dental procedures. The overall TCR incidence was 82.3% (n=70). Operative duration was the strongest predictor of occurrence; each 1-minute increase raised TCR odds by 6.7% (aOR: 1.067, $p < 0.001$).
**Introduction**				
Background/rationale	2	Explain the scientific background and rationale for the investigation being reported	Page 2	-The trigeminocardiac reflex (TCR) is a potent brainstem-

				mediated autonomic response characterized by abrupt bradycardia, asystole, and hypotension following stimulation of trigeminal nerve branches.<br>-While extensively documented in neurosurgical and craniofacial procedures, TCR remains underrecognized in routine dental interventions, particularly in pediatric patients under general anesthesia.
Objectives	3	State specific objectives, including any prespecified hypotheses	End of Introduction	The study objectives are clearly stated, including prespecified hypotheses regarding procedural, anatomical, and temporal predictors of TCR occurrence, severity, and recovery dynamics.
**Methods**				
Study design	4	Present key elements of study design early in the paper	Page 3	This was a prospective observational cohort study conducted in pediatric patients undergoing dental procedures under standardized general anesthesia.
Setting	5	Describe the setting, locations, and relevant dates, including periods of recruitment, exposure, follow-up, and data collection	Page 3	The study was conducted at Kırıkkale University, Faculty of Dentistry, Anesthesiology Clinic, between 13 October

				2025 and 27 March 2026. Data collection was performed during routine pediatric dental procedures under general anesthesia.
Participants	6	(*a*) *Cohort study*—Give the eligibility criteria, and the sources and methods of selection of participants. Describe methods of follow-up<br>*Case-control study*—Give the eligibility criteria, and the sources and methods of case ascertainment and control selection. Give the rationale for the choice of cases and controls<br>*Cross-sectional study*—Give the eligibility criteria, and the sources and methods of selection of participants	Page 3-4	Consecutive pediatric patients (Eligible: 2–18 years; Final analyzed cohort: 2–9 years, as no older patients were encountered during the study period.) with ASA I–II status undergoing dental procedures under general anesthesia were included. Patients with cardiac arrhythmias, neurological disorders affecting autonomic reflexes, or those receiving non-standard anesthetic drugs were excluded.
		(*b*) *Cohort study*—For matched studies, give matching criteria and number of exposed and unexposed<br>*Case-control study*—For matched studies, give matching criteria and the number of controls per case	NA	Not applicable (no matching was performed).
Variables	7	Clearly define all outcomes, exposures, predictors, potential confounders, and effect modifiers. Give diagnostic criteria, if applicable	Pages 3-6	TCR occurrence (≥10% HR/MABP decrease), TCR severity (≥20% decrease), operative duration, procedure type, anatomical location, and lingual manipulation were defined as primary variables. Recovery time was defined as

				time to ≥90% baseline heart rate.
Data sources/ measurement	8*	For each variable of interest, give sources of data and details of methods of assessment (measurement). Describe comparability of assessment methods if there is more than one group	*Pages 3-6*	*Measurements were standardized across all patients under identical anesthetic and monitoring conditions, ensuring comparability between groups.*
Bias	9	Describe any efforts to address potential sources of bias	Pages 3-4	To minimize potential sources of bias, several methodological precautions were implemented. Selection bias related to institutional case-mix was addressed by including consecutive pediatric patients meeting predefined eligibility criteria. Referral bias was acknowledged, as older pediatric patients are more commonly managed under local anesthesia in our institution. Measurement bias was reduced through standardized anesthetic protocols and continuous intraoperative monitoring under identical conditions. Detection bias related to high-sensitivity monitoring (≥10% heart rate reduction threshold) was considered, as this approach may increase the observed incidence of subclinical TCR events. Additionally, all

				procedures were performed by a single anesthesiologist to ensure procedural consistency, and only first-case procedures were included to minimize circadian variability.
Study size	10	Explain how the study size was arrived at	Pages 3-4	Sample size was recalculated following a protocol amendment restricting the study to pediatric patients. Sample size was calculated using both regression-based and proportion-based methods, resulting in a minimum required sample size of 85 participants, which was fully achieved in the final cohort.

Continued on next page

Quantitative variables	11	Explain how quantitative variables were handled in the analyses. If applicable, describe which groupings were chosen and why	Pages 6-7	Continuous variables were analyzed using parametric or non-parametric tests depending on distribution. Operative duration was treated as a continuous predictor in regression models.
Statistical methods	12	(*a*) Describe all statistical methods, including those used to control for confounding	Pages 6-7	Multivariable hierarchical logistic regression was used to identify predictors of TCR occurrence.
		(*b*) Describe any methods used to examine subgroups and interactions	Pages 6-7	Generalized Estimating Equations (GEE) were used to analyze repeated TCR episodes and interaction effects.
		(*c*) Explain how missing data were addressed	Page 7	No missing data were observed in the final dataset.
		(*d*) *Cohort study*—If applicable, explain how loss to follow-up was addressed<br>*Case-control study*—If applicable, explain how matching of cases and controls was addressed<br>*Cross-sectional study*—If applicable, describe analytical methods taking account of sampling strategy	NA	Not applicable (no matching or sampling strategy adjustment required).
		(*e*) Describe any sensitivity analyses	Pages 6-7	Model robustness was assessed using sensitivity analyses including AIC, QIC, and multicollinearity diagnostics.
**Results**				
Participants	13*	(a) Report numbers of individuals at each stage of study—eg numbers potentially eligible, examined for eligibility, confirmed eligible, included in the study, completing follow-up, and analysed	Pages 7-8	A total of 85 pediatric patients were included in the final analysis. No patients were lost to follow-up as all procedures were completed intraoperatively under general anesthesia. All enrolled pediatric participants were analyzed. Adult

				patients enrolled prior to protocol amendment were excluded from final analysis.
		(b) Give reasons for non-participation at each stage	Pages 7-8	Adult patients were excluded following protocol amendment prior to final analysis. No eligible pediatric patients were excluded from analysis.
		(c) Consider use of a flow diagram	Figure 1	A CONSORT-style flow diagram is provided in Figure 1.
Descriptive data	14*	(a) Give characteristics of study participants (eg demographic, clinical, social) and information on exposures and potential confounders	Pages 7-8, Table 1	The study included 85 pediatric patients (median age 5 years [IQR 4–6]), undergoing 1,115 dental procedures under general anesthesia. Baseline demographic and procedural characteristics are presented in Table 1.
		(b) Indicate number of participants with missing data for each variable of interest	NA	No missing data were present for the variables included in the final analysis.
		(c) *Cohort study*—Summarise follow-up time (eg, average and total amount)	NA	Not applicable, as this was an intraoperative observational study with data collected during a single anesthetic episode per patient.
Outcome data	15*	*Cohort study*—Report numbers of outcome events or summary measures over time	*Pages 7-8, Table 1*	*A total of 109 trigeminocardiac reflex (TCR) episodes were observed in 70 patients (82.3% incidence). Of these, 41 were isolated heart rate decreases, 34 were combined heart rate and blood pressure decreases, and 34*

				were classified as severe requiring pharmacologic intervention.
		Case-control study—Report numbers in each exposure category, or summary measures of exposure		
		Cross-sectional study—Report numbers of outcome events or summary measures		
Main results	16	(*a*) Give unadjusted estimates and, if applicable, confounder-adjusted estimates and their precision (eg, 95% confidence interval). Make clear which confounders were adjusted for and why they were included	Pages 7-9	Operative duration was the strongest independent predictor of TCR occurrence (aOR: 1.067, 95% CI: 1.03–1.11, $p < 0.001$). In adjusted models, each 1-minute increase in operative time was associated with a 6.7% increase in odds of TCR. Pulpal involvement increased odds of severe TCR by 3.37-fold (95% CI: 1.19–9.94).
		(*b*) Report category boundaries when continuous variables were categorized	Pages 7-9, Tables 2-3-4-5	Continuous variables (e.g., operative duration) were analyzed without categorization in regression models. TCR severity was categorized as mild (10–20% decrease) and severe (≥20% decrease).
		(*c*) If relevant, consider translating estimates of relative risk into absolute risk for a meaningful time period	NA	Not applicable, as outcomes were intraoperative physiological responses rather than long-term clinical events.

Continued on next page

Other analyses	17	Report other analyses done—eg analyses of subgroups and interactions, and sensitivity analyses	Pages 7-9	Additional analyses included subgroup and interaction models to evaluate procedural and temporal effects on trigeminocardiac reflex (TCR) dynamics. Generalized Estimating Equations (GEE) were used to assess repeated TCR episodes and interaction effects between procedure type and timing. Ordinal logistic regression was applied for frequency-based outcomes, and sensitivity analyses were performed using model diagnostics (AIC, QIC, and multicollinearity checks via VIF) to ensure robustness of findings. No additional post-hoc analyses beyond the predefined statistical plan were conducted.
**Discussion**				
Key results	18	Summarise key results with reference to study objectives	Pages 14-16	This prospective cohort study demonstrates that trigeminocardiac reflex (TCR) in pediatric dental anesthesia is a time- and stimulus-dependent autonomic phenomenon. Operative duration emerged as the strongest predictor of TCR occurrence and frequency, while pulpal involvement significantly increased severity. In contrast, reflex severity was independent of

				surgical duration, indicating dissociation between trigger exposure and response magnitude.
Limitations	19	Discuss limitations of the study, taking into account sources of potential bias or imprecision. Discuss both direction and magnitude of any potential bias	Page 19	The lack of objective depth-of-anesthesia monitoring (e.g., BIS) and the single-center nature of the study are acknowledged. Future multicenter trials incorporat-ing real-time EEG-based monitoring are warranted to validate the "Autonomic Cliff" across broader populations.
Interpretation	20	Give a cautious overall interpretation of results considering objectives, limitations, multiplicity of analyses, results from similar studies, and other relevant evidence	Pages 15-19	The findings should be interpreted in the context of the study’s observational design and multiple statistical comparisons. The observed “Autonomic Cliff” and “Trigger Mass” concepts represent data-driven constructs requiring external validation. While results are consistent with prior evidence of trigeminal autonomic reflexes in surgical settings, direct comparisons are limited due to differences in anesthetic technique, population, and diagnostic thresholds. Overall, the results support a model in which TCR is primarily driven by stimulus intensity and cumulative operative exposure rather than patient demographics.

Generalisability	21	Discuss the generalisability (external validity) of the study results	Pages 15-19	The results are most applicable to pediatric patients undergoing dental procedures under standardized sevoflurane-based general anesthesia in controlled hospital settings. Caution should be exercised when extrapolating findings to adult populations, outpatient sedation, or procedures performed under local anesthesia. While trigeminal-brainstem reflex mechanisms are physiologically well established, their clinical expression may vary across anesthetic techniques and patient populations, limiting direct generalisability of the observed findings.
**Other information**				
Funding	22	Give the source of funding and the role of the funders for the present study and, if applicable, for the original study on which the present article is based	Page 20	This research received no external funding

*Give information separately for cases and controls in case-control studies and, if applicable, for exposed and unexposed groups in cohort and cross-sectional studies.

Note: An Explanation and Elaboration article discusses each checklist item and gives methodological background and published examples of transparent reporting. The STROBE checklist is best used in conjunction with this article (freely available on the Web sites of PLoS Medicine at http://www.plosmedicine.org/, Annals of Internal Medicine at http://www.annals.org/, and Epidemiology at http://www.epidem.com/). Information on the STROBE Initiative is available at www.strobe-statement.org.
