# Supplementary material for: Trigemino-Vagal Recalibration in Pediatric Anesthesia: A Prospective Cohort Study on the “60-Minute Autonomic Cliff,” “Trigger Mass,” and Recovery Dynamics in 1115 Dental Procedures
Source: J Clin Med. 2026 May 8;15(10):3606. doi: 10.3390/jcm15103606 (PMC13207552; doi:10.3390/jcm15103606)
Supplement: Supplementary file 1 [file jcm-15-03606-s001.zip › Supplementary_Material_Table S1.pdf]

Table S1. Statistical Analysis of TCR Severity Across Anatomical Sites (*n*=109)

Anatomical Variable	Mild (isolated HR) (*n*=41)	Mild (combined HR+BP) (*n*=34)	Severe (*n*=34)	*p*-value	Cramer's V
**Dental Arch Axis**				0.115[a]	0.216
Anterior (*n*=14)	3 (21.4%)	8 (57.1%)	3 (21.4%)		
Premolar/Molar (*n*=95)	38 (40.0%)	26 (27.4%)	31 (32.6%)		
**Jaw Axis**				0.936[b]	0.035
Maxilla (*n*=55)	20 (36.4%)	18 (32.7%)	17 (30.9%)		
Mandible (*n*=54)	21 (38.9%)	16 (29.6%)	17 (31.5%)		

[a] Calculated using Fisher's Exact Test (due to n < 5 in certain cells).

[b] Calculated using Pearson's Chi-Square Test.

Cramer's V values indicate effect size: ~0.1 (weak), ~0.3 (moderate).

Statistical Note: The severity of the Trigemino-vagal Reflex (TCR) was categorized into three grades based on hemodynamic response: Mild (isolated HR): isolated HR drop ≥10%, Mild (combined HR+BP): combined HR and BP drop ≥10%, and Severe (HR drop ≥20% with/without ≥20% BP drop).

Statistical comparisons across anatomical sites were performed using Fisher's Exact Test for the dental arch axis (due to small cell frequencies, n < 5) and Pearson's Chi-squared Test (χ^2) for the jaw axis. To determine the clinical magnitude of these associations, Cramer's V was calculated and interpreted as: ~0.1 (small), ~0.3 (medium), and ~0.5 (large).

For the anterior-posterior axis, although formal statistical significance was not reached ($p = 0.115$), a moderate effect size (Cramer's V = 0.216) was observed, indicating a notable clinical trend toward higher severity in molar/premolar sites. The lack of $p < 0.05$ is likely attributable to the limited sample size of the anterior group (*n*=14), a common constraint in prospective pediatric cohorts, rather than a lack of clinical effect. Conversely, the jaw axis showed nearly identical severity distribution ($p = 0.936$, V = 0.035), suggesting that TCR severity is independent of the maxillary or mandibular location.
